# Acute Cardiometabolic Responses to Multi-Modal Integrative Neuromuscular Training in Children

**DOI:** 10.3390/jfmk4020039

**Published:** 2019-06-24

**Authors:** Avery D. Faigenbaum, Jie Kang, Nicholas A. Ratamess, Anne C. Farrell, Mina Belfert, Sean Duffy, Cara Jenson, Jill Bush

**Affiliations:** Department of Health and Exercise Science, The College of New Jersey, Ewing, NJ 08628, USA

**Keywords:** Heart rate, interval training, metabolism, oxygen consumption, physical activity, resistance training, strength training, youth

## Abstract

Integrative neuromuscular training (INT) has emerged as an effective strategy for improving health- and skill-related components of physical fitness, yet few studies have explored the cardiometabolic demands of this type of training in children. The aim of this study was to examine the acute cardiometabolic responses to a multi-modal INT protocol and to compare these responses to a bout of moderate-intensity treadmill (TM) walking in children. Participants (*n* = 14, age 10.7 ± 1.1 years) were tested for peak oxygen uptake (VO_2_) and peak heart rate (HR) on a maximal TM test and subsequently participated in two experimental conditions on nonconsecutive days: a 12-min INT protocol of six different exercises performed twice for 30 s with a 30 s rest interval between sets and exercises and a 12-min TM protocol of walking at 50% VO_2_peak. Throughout the INT protocol mean VO_2_ and HR increased significantly from 14.9 ± 3.6 mL∙kg^−1^∙min^−1^ (28.2% VO_2_ peak) to 34.0 ± 6.4 mL∙kg^−1^∙min^−1^ (64.3% VO_2_ peak) and from 121.1 ± 9.0 bpm (61.0% HR peak) to 183.5 ± 7.9 bpm (92.4% HR peak), respectively. While mean VO_2_ for the entire protocol did not differ between INT and TM, mean VO_2_ and HR during selected INT exercises and mean HR for the entire INT protocol were significantly higher than TM (all *P*s ≤ 0.05). These findings suggest that INT can pose a moderate to vigorous cardiometabolic stimulus in children and selected INT exercises can be equal to or more metabolically challenging than TM walking.

## 1. Introduction

A growing number of children and adolescents fail to accumulate at least 60 min of moderate to vigorous physical activity (MVPA) daily [1]. The far-reaching consequences of physical inactivity during childhood and adolescence are a constellation of cardiometabolic, musculoskeletal, and psychosocial risk factors and diseases that are challenging to manage, difficult to treat and costly to individuals and society [2]. Current efforts to increase MVPA in youth with targeted interventions have had only a small effect [3]. Notably, the impact of walking interventions on physical activity behaviors and health-related fitness measures in school-age youth have been limited [4,5,6]. While walking is a natural form of physical activity that is practical and inexpensive, other types of exercise may be needed to enhance cardiometabolic health, target neuromuscular deficiencies, and increase participation in MVPA. The importance of integrating different types of resistance exercise into youth fitness programs has become particularly important in light of secular declines in measures of muscular strength and power in modern day youth [7,8]. The available evidence supports a link between muscular fitness and physical activity, particularly vigorous intensity physical activity, in children and adolescents [9].

Integrative neuromuscular training (INT) is a type of exercise characterized by intermittent bouts of different strength- and skill-building exercises that are designed to improve fundamental movement skills, increase muscular fitness and prepare participants for exercise and sport activities [10]. INT has emerged as an effective strategy for improving health- and skill-related components of physical fitness in school-age youth, [11,12,13], and limited evidence suggests that this type of training may also offer cardiometabolic benefits [14,15]. Previous studies investigating the effects of INT on children have found significant improvements in sprinting, running, jumping, throwing and lifting performance following 8 to 10 weeks of training [11,12,16]. However, it is also important to examine the acute cardiometabolic responses to INT because the amount of time youth spend in vigorous physical activity is more strongly associated with positive health outcomes than light or moderate-intensity physical activity [17,18,19]. An analysis of accelerometer data from a large sample of children found vigorous physical activity was strongly associated with metabolic health whereas associations of light to moderate physical activity were weak to moderate [18]. While research evidence supports the safety, efficacy and feasibility of INT for children [11,12,16], the acute cardiometabolic responses to INT are poorly understood.

Researchers examined the acute cardiometabolic responses to a single-mode interval training protocol with medicine balls [14] or battling ropes [15] in children and found that this type of exercise can pose a potent cardiometabolic stimulus. For example, mean heart rate (HR) and oxygen uptake (VO_2_) values during a 10-min bout of medicine ball interval training ranged from 61.1% to 89.6% of HR peak and from 28.2% to 63.5% of VO_2_ peak [14]. Similarly, others found that the acute responses to a 12-min session of resistance training or intermittent noncontact boxing in early adolescents could be characterized as “vigorous” and therefore contribute to daily moderate to vigorous physical activity (MVPA) recommendations [20]. Due to the increasing interest in high-intensity interval training and the potential for multi-modal INT to modulate disease risk factors and improve health outcomes in youth [10,21,22], there is strong rational to further examine the acute cardiometabolic responses to INT in youth. Of relevance to the current study, strategic efforts to strengthen and improve physical education and physical activity opportunities with novel and time efficient exercise interventions are needed to increase MVPA in school-age youth and foster a healthy generation [23].

To the authors’ knowledge, no previous study has examined the acute cardiometabolic responses to multi-modal INT in children and direct comparisons between INT and traditional exercise interventions such as walking have not been reported. While brisk walking can offer health benefits for children, INT has been found to enhance cardiometabolic health and neuromuscular fitness in youth [11,12,13]. Additional research on the acute responses to INT could be used to establish preliminary cardiometabolic references values for INT and inform the design of novel exercise interventions for children. Therefore, the purpose of this study was to examine the acute cardiometabolic responses to a multi-modal INT protocol in children and to compare these responses to a bout of moderate-intensity treadmill walking. Based on previous findings regarding the acute cardiometabolic responses to different exercise modalities in youth [14,15], we hypothesized that selected INT exercises would elicit a cardiometabolic response that was equal to or greater than brisk walking in children.

## 2. Materials and Methods

### 2.1. Participants

A convenience sample of 14 healthy children (8 boys and 6 girls; mean ± SD age 10.7 ± 1.1 years; height 143.2 ± 6.8 cm and body mass 36.3 ± 9.9 kg) volunteered to participate in this study. Participants were active members of local sports teams (primarily soccer and lacrosse), but none participated regularly in resistance training. Parents completed a modified physical activity readiness questionnaire to evaluate the health status of the participants and assess the safety for performing vigorous exercise. All parents signed a parental permission form and all participants signed a child assent form and were informed of the benefits and risks of this investigation. This study was approved by the Institutional Review Board at The College of New Jersey (process No: 2017-0080, 7 February 2018).

### 2.2. Peak Aerobic Capacity Testing

All participants reported to the Human Performance Laboratory at least 2 h postprandial for peak aerobic capacity testing. VO_2_ peak was assessed using the Fitkids treadmill test protocol [24] and a metabolic system (MedGraphics ULTIMA Metabolic System, MedGraphics Corporation, St. Paul, MN, USA). The Fitkids treadmill test is a valid and reproducible exercise test for children that consists of 90 s stages with incremental increases in speed and incline until volitional exhaustion [24]. Breath-by-breath VO_2_ data were obtained and VO_2_ peak was determined by recording the highest measure observed during the test [25]. HR was monitored using a soft chest strap with a HR sensor (Model A300; Polar Electro Inc., Woodbury, NY, USA). HR peak was defined as the highest value achieved during the test. Participants were asked to manually signal without verbalizing their rating of perceived exertion (RPE) during the test [26]. Prior to testing, height was measured to the nearest 0.1 cm using a wall-mounted stadiometer and body mass was measured to the nearest 0.5 kg using an electronic scale. For both measurements, participants wore light cloths and no shoes.

### 2.3. Integrative Neuromuscular Training Protocol

Participants returned to the Human Performance Laboratory to perform the INT protocol within 2 to 7 days of the peak aerobic capacity test. The INT protocol used in this study was based on previous pediatric research [11,14,15,27] and included strength- and skill-building exercises that were appropriate for children [10]. Our INT protocol consisted of the following six exercises: (1) balance board squats (EX1; 15 repetitions), (2) medicine ball squats with toss and catch (EX2; 15 repetitions), (3) BOSU™ planks with side steps (EX3; 20 repetitions), (4) medicine ball forward lunges (EX4; 16 repetitions), (5) battling rope double arm waves (EX5; 30 repetitions) and medicine balls slams (EX6; 15 repetitions). The 6 INT exercises were performed in successive order with each exercise interval lasting 30 s in duration. Each exercise was performed twice with a rest interval of 30 s in between sets and exercises. The 12 time intervals corresponding to the initiation and termination of each 30 s INT set were carefully monitored and labeled. The total duration of the INT protocol was 12 min (including 30 s recovery following the last exercise).

Pilot testing from our center found that a 2.3 kg medicine ball and a 4.1 kg battling rope were appropriate for children. Participants were asked to follow a specific cadence using a metronome and to try and complete a target number of repetitions during each set. A research assistant performed the INT protocol at the desired cadence with each participant during each set and provided a quick review of the upcoming exercise during each rest interval. All participants performed the same exercises in the same order. Participants were asked to manually indicate their RPE after each INT set on a visually presented scale consisting of verbal expressions with a numerical response range of 0 to 10 and five pictorial descriptors that represent a child at varying levels of exertion [26]. Participants became familiar with the INT protocol during a familiarization session which took place after the peak aerobic capacity test. During the familiarization session participants practiced each INT exercise and proper technique was reinforced with exercise-specific coaching cues.

### 2.4. Treadmill Protocol

Participants performed the TM trial 2 to 7 days after the INT protocol. The TM protocol used in this study was designed to be a moderate-intensity walking protocol that is consistent with general physical activity recommendations for school-age children [28,29]. Participants walked briskly at a predetermined exercise intensity of 50% VO_2_ peak for 12 min. The speed and grade of the treadmill were adjusted to maintain the desired exercise intensity throughout the 12-min session. Cardiometabolic data were collected during the same 30 s time intervals as INT. Participants were asked to manually indicate their RPE during the TM trial [26].

### 2.5. Experimental Measurements: Oxygen Uptake and Heart Rate

VO_2_ and HR were measured at rest and throughout the INT and TM protocols following similar experimental procedures. On arrival, each participant was asked to drink water *ad libitum* to prehydrate and was fitted with the same child-size respiratory mask and heart rate monitor used for the maximal aerobic capacity test. HR data were downloaded for analysis using a computer software program. HR data analyzed were the mean values collected during each 30-s time interval throughout the 12-min INT and TM protocols. Breath-by-breath VO_2_ was measured during the INT and TM protocols using the same metabolic system used for maximal aerobic capacity testing. Values for relative VO_2_, minute ventilation (V_E_) and respiratory exchange ratio (RER) were recorded during the entire protocol. Individual breath-by-breath data points for all metabolic variables were averaged for each 30 s interval. Prior to each trial, each participant sat quietly in a chair for 5 min to collect baseline data. Once complete, the researcher briefly reviewed session instructions. Subsequently, each participant performed 2 to 3 min of calisthenics (e.g., arm circles and knee lifts) prior to INT or 2 to 3 min of low intensity walking prior to TM. Verbal encouragement was provided throughout the INT and TM trials.

### 2.6. Statistical Analysis

Descriptive statistics (mean ± SD) were calculated for all dependent variables. For each protocol, the mean values for VO_2_, V_E_, RER, and HR were averaged every 30 s in time-match intervals as well as for the entire 12-min protocol. A 2 (INT or TM) × 12 (time interval sets) analysis of variance with repeated measures was used to analyze within and between participant cardiometabolic and RPE data. A significant F ratio was followed by pairwise comparisons to detect differences between INT and TM at a given time interval using Bonferroni’s adjustments. In addition, a dependent *t*-test was used to compare mean VO_2_, V_E_, RER, and HR of the entire protocol between INT and TM. For all statistical tests, a probability level of *p* < 0.05 denoted statistical significance. Statistical analyses were conducted in SPSS (version 24; SPSS, Chicago, IL, USA).

## 3. Results

**Figure 1:**
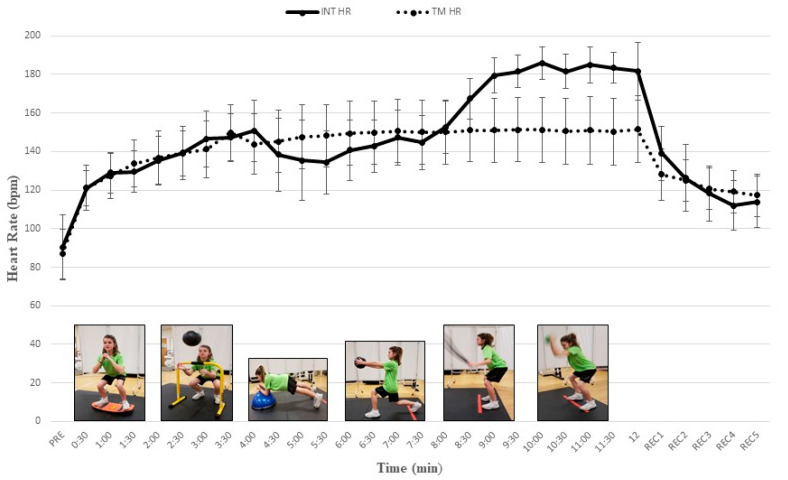
Heart rate (HR) responses (mean ± SD) during integrative neuromuscular training (INT) and treadmill (TM) protocols. PRE = Baseline; REC = recovery. See Table 1 for significant differences between INT exercises and protocols.

**Figure 2:**
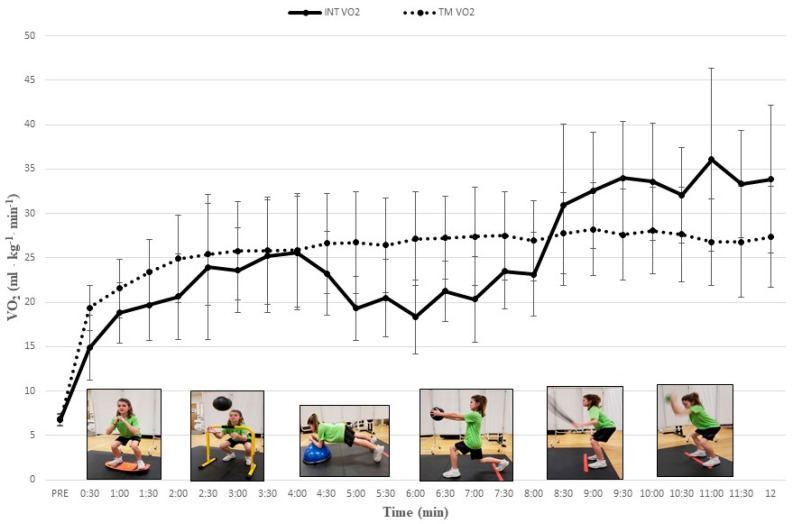
Relative oxygen uptake responses (mean ± SD) during integrative neuromuscular training (INT) and treadmill (TM) protocols. PRE = Baseline; See Table 1 for significant differences between INT exercises and protocols.

All participants completed study procedures and no injuries or unexpected events occurred. Our post hoc comparisons revealed a progressive increase in cardiometabolic demand as VO_2_, V_E_, RER, and HR increased significantly throughout our multi-exercise INT protocol. During the INT protocol mean HR significantly increased from 121.1 ± 9.0 b∙min^−1^ to 183.5 ± 7.9 b∙min^−1^ and mean VO_2_ significantly increased from 14.9 ± 3.6 mL∙kg^−1^∙min^−1^ to 34.0 ± 6.4 mL∙kg^−1^∙min^−1^ (Table 1). Values for HR, V_E_, and RER tended to increase with each successive INT exercise and paralleled VO_2_ data. Mean VO_2_ and HR during EX5 and EX6 of the INT protocol were significantly higher than during time-matched TM intervals (Table 1) (all *Ps* < 0.05). Figure 1; Figure 2 depict the gradual increase in HR and VO_2_, respectively, during the INT protocol as compared to TM.

The relative cardiometabolic intensity of each INT exercise (expressed as a percentage of values attained during maximal aerobic capacity testing) ranged from 61.0% to 92.4% for HR and from 28.2% to 64.3% for VO_2_. The relative cardiometabolic demands of each INT exercise compared to time-matched TM time intervals are outlined on Table 2. The significant increases in cardiometabolic responses during INT mirrored significant increases in RPE. The mean RPEs (out of 10) for INT EX1 to EX6 were 1.14 ± 0.85, 2.26 ± 0.98, 2.91 ± 1.04, 3.55 ± 1.23, 5.42 ± 1.45 and 6.68 ± 1.67, respectively. There was no significant difference in mean VO_2_ between the entire 12-min INT and TM protocols; however, mean values for HR, V_E_ and RER for the entire 12-min protocol were significantly higher during INT than TM (all *Ps* < 0.05) (Table 3). The mean VO_2_ throughout the 12-min TM protocol was 49.5% of VO_2_ peak attained during maximal aerobic capacity testing.

## 4. Discussion

The aim of our study was to examine the acute cardiometabolic responses to a multi-modal INT protocol in children and to compare these responses to a bout of moderate-intensity TM walking. Consistent with our hypothesis, we found a progressive, multi-modal INT protocol comprising 30 s of work with 30 s of passive recovery can pose a moderate to vigorous cardiometabolic stimulus in children and selected INT exercises can be equal to or more metabolically challenging than TM walking. While other pediatric investigations detailed the acute physiological responses to intermittent bouts of single-mode exercise with cycling, sprinting, medicine balls or battling ropes [14,15,30,31], this is the first study to describe the acute cardiometabolic demands of a mixed-exercise INT protocol in children. No significant differences were observed in mean VO_2_ between the entire 12-min INT and TM protocols. However, mean HR, V_E_, and RER were significantly higher during INT than TM. Given the link between vigorous physical activity and positive health outcomes in youth [17,18,19], our findings provide insight into the potential cardiometabolic benefits of INT if performed at the requisite weekly frequency.

Participants in our study were physically active children with a peak aerobic capacity of 52.9 ± 9.4 mL⋅kg^−1^⋅min^−1^ and a peak HR of 198.5 ± 5.5 bpm. During the INT protocol, VO_2_ increased from 14.9 ± 3.6 to 34.0 ± 6.4 mL⋅kg^−1^⋅min^−1^ and HR increased from 121.1 ± 9.0 bpm to 183.5 ± 7.9 bpm (Table 1). As shown on Table 2, the relative intensity of each INT exercise expressed as a percentage of VO_2_ peak and HR peak ranged from 28.2% to 64.3% and from 61.0% to 92.4%, respectively. The mean VO_2_ and HR responses during the entire 12 min INT protocol were 49.5% and 73.1%, respectively, of peak values. When compared to a standard classification of physical activity intensity based on percentage of VO_2_ peak or HR peak, our findings indicate that the overall intensity of our 12-min INT protocol could be characterized as “moderate” (i.e., 46–63% VO_2_ peak and 64–76% HR peak) whereas the intensity of individual INT exercises could be characterized as “light”, “moderate”, or “vigorous” depending upon the mode and complexity of each movement [32]. Knowing the intensity levels of different INT exercises can help researchers and practitioners design interventions that optimize training-induced adaptations and encourage compliance in all participants.

The progressive increase in HR throughout our INT protocol was consistent with other reports that examined the acute physiological responses to different modes of resistance exercise in youth [14,15,20]. During a 10-min bout of medicine ball interval training (30 s/exercise and 30 s rest/set) researchers reported that mean HR values during work sets ranged from 121.5 ± 12.3 bpm (61.1% HR peak) to 178.3 ± 9.4 bpm (89.6% HR peak) [14]. Harris and colleagues characterized the acute responses to resistance exercise and HIIT in early adolescents (12–13 years) and reported mean HR over all 12 work sets of 169.9 ± 9.2 bpm for resistance training and 179.0 ± 5.6 bpm for HIIT which represented 85% and 90% of HR peak, respectively [20]. In our study, the gradual increase in HR from 61% HR peak to over 90% HR peak was expected because the structure of our multi-modal INT protocol included six different exercises that progressed from a less intense squatting exercise to a more explosive movement. These findings are notable because high-intensity interval training characterized by short bouts of vigorous intensity activity may be needed to elicit the greatest improvements in cardiometabolic health and aerobic fitness in children [21,33]. Of interest, participants in our study recovered quickly from the demands of INT as evidenced by heart rates of 139.1 ± 13.9 bpm, 118.4 ± 14.2 bpm, and 113.9 ± 13.5 bpm after 1, 3 and 5 min of recovery, respectively (Figure 2). These observations are consistent with others who reported a faster post-exercise recovery heart rate in children than endurance adult athletes. [34].

The findings related to relative VO_2_, V_E_ and RER build upon previous reports investigating different modes of youth resistance training. During a progressive interval protocol with 5 battling rope exercises, relative VO_2,_ V_E_ and RER increased to 30.0 mL⋅kg^−1^⋅min^−1^ (64.8% VO_2_ peak), 40.8 L/min and 1.07, respectively [15]. In another report, relative VO_2,_ V_E_ and RER reached 34.9 mL⋅kg^−1^⋅min^−1^ (63.6% VO_2_ peak), 40.4 L/min and 0.95, respectively, during medicine ball interval training [14]. While Baquet and colleagues reported mean VO_2_ values of 35.5 mL⋅kg^−1^⋅min^−1^ (64.6% VO_2_ peak) to 47.0 mL⋅kg^−1^⋅min^−1^ (85.9% VO_2_ peak) during sprint high intensity interval exercise in children [31], differences in exercise characteristics, workload duration and rest interval length can explain, at least in part, these findings. The progressive increase in RER that reached values above 1.0 which were greater than those observed during TM walking further attested to the intense nature of our INT protocol. Collectively, it appears that INT and other types of interval training could be used to bring about positive cardiometabolic adaptations in children.

Our findings demonstrate that INT characterized by short exercise intervals interspersed with brief rest intervals can pose a moderate to vigorous cardiometabolic stimulus in youth. Unlike brisk walking, INT typically requires the whole body to function as a unit in order to perform movements proficiently with proper technique at the desired cadence. During our multi-modal INT protocol, the highest VO_2_ and HR values were achieved during EX5 (battling rope double arm wave) and EX6 (medicine ball slams), respectively. Both of these exercises require a substantial involvement of the upper and lower body since participants vigorously waved a 4.1 kg battling rope with both arms or forcibly slammed a 2.3 kg medicine ball against the floor at maximal or near maximal velocity. Interestingly, Ratamess and colleagues examined the acute cardiometabolic response to 13 different resistance exercise protocols in adults and found that the battling rope double arm wave elicited the highest responses [35]. While the greater complexity and muscle mass activation of the INT exercises towards the end of our protocol suggest that the choice of exercise is a primary determinant of the cardiometabolic responses to INT, the cumulative effects of fatigue and cardiovascular drift during our INT protocol should also be considered because the cardiometabolic responses to each INT exercise were likely influenced by the subsequent fatigue from the previous exercise.

The structure of our INT protocol was based on previous fitness interventions with children and included 2 sets of 6 different exercises with a 30 s rest interval in between sets and exercises [11,27]. Due to the age of the participants and the relative intensity of selected INT exercises, a progressive, multi-modal INT protocol with passive recovery intervals was arguably required to maintain safety, motivation and adherence. Sustained bouts (>10 min) of physical activity are rare in children and the natural tempo of their activity is characterized by short bursts lasting a few seconds [36]. Furthermore, it is important to note that the objective of our investigation was not to disentangle the acute demands of each INT exercise, but rather to examine the acute cardiometabolic responses to a novel INT protocol and to compare these responses to a more traditional form of continuous physical activity of comparable time and overall VO_2_. Notably, we used a time period of 12 min so that the protocol could be readily incorporated into a physical education class or sports practice. With that said, the results from our investigation provide preliminary cardiometabolic reference values for a multi-modal INT protocol and highlight the versatility of INT because different exercises could be combined to offer light, moderate, vigorous or variable intensity training depending on the needs, goals and abilities of the children.

Our findings support the integration of INT into school- and community-based youth fitness programs and inform the development of interventions aimed at increasing time spent in MVPA. The progressive increase in cardiometabolic intensity throughout the INT protocol was consistent with the participants perceptions as evidenced by significant increases in RPE. Others found that youth could rate their perceived exertion during resistance training and our observations support the use of RPE to monitor the intensity of INT in children [20,37]. We also observed that our INT protocol was challenging and appealing for the participants. This was evidenced by 100% compliance with research instructions and testing protocols. Although the affective response to INT were not explored in our investigation, Malik and colleagues found that enjoyment was higher following high-intensity interval exercise compared with continuous moderate-intensity exercise in adolescents [38]. Given that a child’s level of enjoyment is a strong predictor of physical activity participation [39], further examination of the affective responses to INT are warranted.

The INT exercises used in our investigation were intended to progress from less intense to more intense. EX1 consisted of squatting on a balance board at a controlled cadence and EX6 required participants to repeatedly slam a 2.3 kg medicine ball against the floor with energy and vigor. These are important considerations when discussing our findings because the cardiometabolic responses to INT are dependent upon various factors including the intensity of muscle actions, type of muscle actions, the amount of muscle mass used, rest intervals and body position [14,15,40]. In addition, age, fitness level and body mass index can influence the acute cardiometabolic responses to exercise and the kinetics of recovery after exercise [25,34,41]. Children appear to be less susceptible to neuromuscular fatigue than adults following resistance training [42] and the post-exercise decline in VO_2_ seems to be faster in children with a higher peak VO_2_ than those with a lower peak VO_2_ [34,43]. Thus, the results of our investigation should be interpreted within the context of a mixed model exercise approach that reflects how children may actually perform INT during physical education or sports practice.

We acknowledge that the maturity status of the participants was not assessed and therefore we were unable to determine if all participants were prepubertal. Also, the participants in our study were active, healthy girls and boys so the homogeneity of our sample limits generalizability to other populations including those with illnesses or disabilities that alter movement or mechanical efficiency. It is also important to consider the design of our INT protocol and the limited INT experience of our participants. Acute program variables will impact the cardiometabolic responses to INT and as children become more skilled and efficient at performing INT exercises the acute cardiometabolic demands to a given protocol will not be constant. While other pediatric researchers examined the acute cardiometabolic responses to single-mode exercise, the design of our progressive, multi-modal INT protocol arguably provides greater translatability to school- and community-based programs in which the physical fitness levels and activity interests of children can vary widely.

## 5. Conclusions

INT has been investigated as a potentially potent and time efficient method of enhancing neuromuscular fitness in youth [11,12,16] and our novel findings suggest that this innovative training method consisting of strength- and skill-building exercises could also provide a sufficient training stimulus needed to induce cardiometabolic adaptations. Considering the amount of time children spend in MVPA during physical education and youth sport practice is falling short of expectations [44,45], INT could be a worthwhile addition to school- and community-based programs to target exercise deficits. Unlike continuous bouts of moderate-intensity walking, a progressive INT intervention with battling rope and medicine ball exercises may better prepare youth for the vigorous intensity nature of game and sport activities. These observations have practical relevance for teachers, coaches and health care providers who design exercise programs and sport practices for children. Altogether, the acute cardiometabolic responses to INT along with high compliance to our study procedures provide support for future training studies to better understand the multidimensional benefits of INT on health, fitness and performance in youth.

## Figures and Tables

**Table 1:** Cardiometabolic responses during integrative neuromuscular training (INT) and treadmill (TM) walking.

Interval (min)	INT EX/set	VO_2_		V_E_		HR		RER	
		INT	TM	INT	TM	INT	TM	INT	TM
1 (0–0.5)	1/1	14.9 ± 3.6 ^b−l^	19.2 ± 2.5	14.9 ± 4.5 ^b−l^	16.8 ± 2.5	121.1 ± 9.0 ^b−d,g−l^	121.1 ± 11.7	0.88 ± 0.07 ^j−l^	0.82 ± 0.05
2 (1.0–1.5)	1/2	19.7 ± 3.9 ^a,d,h−l^	23.4 ± 3.7	19.7 ± 6.6 ^a,d,e,h−l^	20.8 ± 4.5	129.6 ± 10.9 ^a,c−d,g−l^	133.8 ± 12.2	0.88 ± 0.08 ^j−l^	0.85 ± 0.06
3 (2.0–2.5)	2/1	23.9 ± 8.2 ^a,j−l^	25.4 ± 5.7	24.7 ± 7.7 ^a,i−l^	23.8 ± 6.0	139.1 ± 11.6 ^a,b,d,i−l^	139.3 ± 13.7	0.90 ± 0.07 ^j−l^	0.89 ± 0.04
4 (3.0–3.5)	2/2	25.2 ± 6.3 ^a,b,j−l^	25.8 ± 6.6	27.9 ± 9.2 ^a,b,j−l^	24.2 ± 6.6	147.3 ± 12.4 ^a−c,i−l^	146.0 ± 14.5	0.93 ± 0/07 ^j−l^	0.90 ± 0.05
5 (4.0–4.5)	3/1	23.2 ± 4.7 ^a,j−l^	26.6 ± 5.6	26.7 ± 9.5 ^a,b,i−l^	25.4 ± 7.0	138.4 ± 19.0 ^i−l^	145.1 ± 16.1	0.96 ± 0.07 ^j−l^	0.90 ± 0.06
6 (5.0–5.5)	3/2	20.5 ± 4.3 ^a,h−l^	26.4 ± 5.3 *	23.4 ± 9.4 ^a,h−l^	25.0 ± 6.9	134.3 ± 16.5 ^i−l^	148.1 ± 16.2	0.94 ± 0.06 ^j−l^	0.90 ± 0.06
7 (6.0–6.5)	4/1	21.2 ± 3.4 ^a,h−l^	27.2 ± 4.7 *	24.3 ± 9.1 ^a,b,i−l^	25.8 ± 5.8	142.8 ± 13.6 ^a,b,i−l^	150.0 ± 16.4	0.95 ± 0.07 ^j−l^	0.90 ± 0.04
8 (7.0–7.5)	4/2	23.4 ± 4.2 ^a,b,f−l^	27.5 ± 4.9 *	26.7 ± 9.1 ^a,b,f,i−l^	26.6 ± 7.0	144.8 ± 14.0 ^a,b,i−l^	150.1 ± 16.7	0.90 ± 0.05 ^j−l^	0.90 ± 0.05
9 (8.0–8.5)	5/1	30.9 ± 9.1 ^a,b,f,g^	27.8 ± 4.6	38.3 ± 14.2 ^a−c,e−j^	26.7 ± 6.0	167.4 ± 10.5 ^a−h,j−l^	151.1 ± 16.1	0.95 ± 0.08 ^j−l^	0.91 ± 0.05
10 (9.0–9.5)	5/2	34.0 ± 6.4 ^a−h^	27.6 ± 5.1 *	52.2 ± 12.4 ^a−i^	26.4 ± 5.6 *	181.6 ± 8.3 ^a−i, k,l^	151.3 ± 16.7 *	1.15 ± 0.07 ^a−i^	0.90 ± 0.05 *
11 (10–10.5)	6/1	32.0 ± 5.4 ^a−h^	27.6 ± 5.3 *	46.2 ± 10.2 ^a−h^	26.7 ± 5.7 *	181.5 ± 9.0 ^a−i^	150.5 ± 17.2 *	1.12 ± 0.07 ^a−i^	0.90 ± 0.05 *
12 (11–11.5)	6/2	33.3 ± 6.0 ^a−h^	26.8 ± 6.2 *	48.6 ± 11.0 ^a−h^	25.1 ± 6.5 *	183.5 ± 7.9 ^a−i^	150.4 ± 17.3 *	1.07 ± 0.06 ^a−i^	0.89 ± 0.05 *

All values are mean ± SD. EX = exercise, VO_2_ = oxygen uptake, mL∙kg^−1^∙min^−1^; V_E_ = minute ventilation, L/min; HR = heart rate; RER = respiratory exchange ratio. ^a^ vs. interval 1; ^b^ vs. interval 2; ^c^ vs. interval 3; ^d^ vs. interval 4; ^e^ vs. interval 5; ^f^ vs. interval 6; ^g^ vs. interval 7; ^h^ vs. interval 8; ^i^ vs. interval 9; ^j^ vs. interval 10; ^k^ vs. interval 11; ^l^ vs. interval 12. * different than INT. *p* ≤ 0.05.

**Table 2:** Relative cardiometabolic intensity during integrative neuromuscular training (INT) and treadmill (TM) walking intervals.

Interval (min)	INT EX/Set	% VO_2_ Peak		%HR Peak	
		INT	TM	INT	TM
1 (0–0.5)	1/1	28.2	36.3	61.0	61.0
2 (1.0–1.5)	1/2	37.2	44.2	65.3	67.4
3 (2.0–2.5)	2/1	45.1	48.0	70.1	70.1
4 (3.0–3.5)	2/2	47.6	48.8	74.2	73.5
5 (4.0–4.5)	3/1	43.8	50.2	69.7	73.1
6 (5.0–5.5)	3/2	38.7	49.9	67.6	74.6
7 (6.0–6.5)	4/1	40.0	51.4	71.9	75.6
8 (7.0–7.5)	4/2	44.2	52.0	72.9	75.6
9 (8.0–8.5)	5/1	58.4	52.5	84.3	76.1
10 (9.0–9.5)	5/2	64.3	52.2	91.5	76.2
11 (10–10.5)	6/1	60.5	52.2	91.4	75.8
12 (11–11.5)	6/2	62.9	50.7	92.4	75.8

**Table 3:** Mean cardiometabolic responses during the entire 12 min integrative neuromuscular training (INT) and treadmill (TM) protocols.

	INT	TM
VO_2_ (mL∙kg^−1^∙min^−1^)	25.4 ± 4.5	26.2 ± 4.5
V_E_ (l∙min^−1^)	29.79 ± 8.0	24.7 ± 5.5 *
HR (beats∙min^−1^)	153.4 ± 10.6	145.2 ± 15.0 *
RER	0.96 ± 0.04	0.89 ± 0.05 *

All values are mean ± SD. * different than INT. *p* ≤ 0.05.
